# Parameters of Concrete Modified with Micronized Chalcedonite

**DOI:** 10.3390/ma16093602

**Published:** 2023-05-08

**Authors:** Anna Kotwa, Piotr Ramiączek, Paulina Bąk-Patyna, Robert Kowalik

**Affiliations:** 1 Faculty of Civil Engineering and Architecture, Kielce University of Technology, 25-314 Kielce, Poland; p.ramiaczek@tu.kielce.pl (P.R.); pbak@tu.kielce.pl (P.B.-P.); 2 Faculty of Environmental, Geomatic and Energy Engineering, Kielce University of Technology, 25-314 Kielce, Poland

**Keywords:** additive, cement, absorbability, capillary rise, chalcedonite dust, compressive strength

## Abstract

The PN-EN 197-1:2012 standard allows the use of additives as the main component above 5.0% by mass, as well as as a secondary component in an amount less than 5.0% by mass of cement. Proper selection of additives positively affects the rheological characteristics and hardened concrete parameters during longer maturity periods. Additives have already become an integral component of concrete mixes. The aim of the research is to confirm the possibility of using the tested additive in the composition of concrete mixes in an amount of 15% relative to the amount of cement, which would solve the problem of storing and utilizing waste generated during the production of broken chalcedonite aggregates. The planned laboratory tests were carried out for concrete of three classes, C30/37, C35/45, C40/50, according to the PN-EN 206+A1:2016-2 standard, with the addition of chalcedonite dust in a constant amount of 15% relative to cement, and three series without additives as control series. The additive used for concrete mixes was chalcedonite dust with a diameter below 72 μm. It is waste from a broken aggregate mine. The research program included rheological tests of fresh concrete mix, i.e., air content, consistency, bulk density, as well as parameters of hardened concrete mix—compressive strength, absorbability, and capillary uptake. Compressive strength was tested after 7, 14, 28, 56, and 90 days. The laboratory tests aimed to verify whether the addition of 15% chalcedonite dust additive would not worsen the predicted hardened concrete parameters resulting from the designed concrete classes. All three tested series, C30/37, C35/45, and C40/50, with the addition of 15% chalcedonite dust relative to the amount of cement, achieved the assumed strength classes after 28 days of maturation. Concrete mix components were correctly designed. The addition of chalcedonite dust to the concrete mix did not cause a decrease in compressive strength to the extent that the analyzed series did not meet the normative requirements for concrete classes according to the PN-EN 206+A1:2014 standard. The results of absorbability testing indicate water absorption below 5%, while the increase in sample mass in the capillary uptake test gained similar values.

## 1. Introduction

The PN-EN 206+A2:2021-08 standard “Concrete—Requirements, properties, production and conformity” defines an additive as a component of concrete that is highly fragmented [1]. The additive is used to improve certain rheological properties of the concrete mix or hardened concrete [2,3,4,5]. The standard distinguishes between two types of additives: Type I additives are nearly inert (pigments, lime, and quartz meal). They do not react with cement or water and do not affect hydration. Type I additives fill the spaces between cement grains and can also improve structure due to the size, shape, or form of the grains [6,7]. They are often used to improve workability and impermeability. Type I additives are added to concrete mixtures that contain sand with small amounts of fine fractions [8,9,10].

Type II additives are pozzolanic or weakly hydraulic additives [11,12]. Pozzolanic additives can be natural (such as volcanic ash) or artificial (such as fly ash, silica fume, or blast furnace slag). These additives react with calcium hydroxide, which is produced during hydration, and as a result, the concrete hardens Type II additives can be added to concrete to improve specific properties or to obtain special rheological properties [13]. The change in concrete parameters and rheological properties depends on the type of additive, its quantity in the mixture, and its degree of fragmentation [14,15,16]. Additives with established suitability should be introduced into concrete mixtures. Type I and II additives that will be added to the concrete mixture should be tested in preliminary tests. Such tests should be performed for fresh concrete mixtures as well as hardened concrete. As an alternative to preliminary tests, tests performed on other batches with similar mixture compositions or the designer’s many years of experience in concrete mixtures can be considered [17,18,19,20].

Type II additives can be included in the concrete mixture as part of the cement content and the water/cement ratio. The PN-EN 206 standard specifies three concepts for incorporating Type II additives into the concrete mixture:

- The coefficient k concept;
- The equivalent performance concept;
- The equivalent performance combination concept.

The principle of using the k-factor concept mainly involves comparing the compressive strength of concrete without additives, i.e., the reference concrete, with the concrete in which part of the cement has been replaced with a selected additive. The comparative criterion is durability and/or compressive strength. The k-factor concept allows for the use of type II additives with aminimal amount of cement in a given exposure class and the replacement of the w/c factor with the water/(cement + k* additive) ratio [20,21,22].

The concept of equivalent functional properties of concrete is based on the assumption that the properties of the concrete modified with an additive correspond to the properties of the reference concrete. The assumption can be met if we use cement that complies with the requirements of the PN-EN 197-1:2012 standard and also maintains the minimum amount of cement and the maximum w/c ratio in a given exposure class.

The concept of combining equivalent functional properties is based on the assumption of the minimum amount of cement and the maximum w/c factor in a given exposure class. The use of type II additives in concrete mixes is possible only for additives with defined suitability whose origin and characteristics are known, [22,23,24].

Additives used in concrete should meet normative requirements and be used in accordance with the standards. Proper use of additives can improve the rheological properties of the concrete mix and the parameters of the hardened concrete, as well as allow the utilization of waste material heaps [25,26,27]. One of the waste materials is chalcedonite dust from the Inowłodz mine, the Teofilów deposit.

Micronized chalcedonite is a product that contains respirable quartz. It is classified as STOT REI according to the criteria set out in Regulation (EC) No. 1272/2008. Depending on the atmospheric conditions and storage methods, the dust containing free crystalline silica can be suspended in the air. The respirable fraction of chalcedonite dust, when inhaled by humans for a prolonged period, can cause upper respiratory tract inflammation;in a drastic situation of repeated respiratory diseases over several years, it can lead to diffuse nodular fibrosis of lung tissue, commonly called silicosis. The main symptoms of silicosis in humans are persistent coughing, difficulty breathing, pulmonary insufficiency, and worsening apnea. Inhalation of crystalline silica dust should be prohibited. The dust should be handled with extreme caution to avoid inhalation [28].

Therefore, a significant challenge is the problem of utilizing waste materials generated during the production of crushed stone in Poland. This is about chalcedonite dust, whose main component is SiO_2,_ with the EC number 238-878-4. It also contains trace amounts of aluminosilicates and metal compounds, as well as quartz, opal, iron hydroxides, pyrite, manganese compounds, and clay minerals. From a chemical point of view, chalcedonite is a homogeneous material composed of about 94% silica by weight. Utilizing billions of tons of waste material fits into the protection of the natural environment [28,29]. Using the accumulated amounts of waste material would allow less clinker consumption, reduce emissions of air and soil pollutants, save natural raw materials, clean up the areas around the mines, and protect people living in the near vicinity from respiratory diseases [30,31,32].

The studies carried out so far indicate that the introduction of 20% of chalcedonite dust into the concrete mix results in a decrease in compressive strength after 28 days by up to 15%. Replacing the cement with a 15% chalcedonite additive reduces the strength after 28 days of curing by 10%. Reducing the addition to 5% in the concrete mix does not reduce the compressive strength.

The addition of 20% chalcedonite dust in the capillary rise test results in weight gain after 28 days of maturation by up to 54%. In the capillary rise test, it can be seen that the weight gain is comparable for the tested series, in which the additive was used in the amount of 5% and 10% and was about 37 kg/m^2^ after 28 days of curing.

On the other hand, water absorption after 90 days of maturation with 20% of chalcedonite dust is lower by about 11% compared to the series of concretes without the addition. Absorption after 28 days of maturation is lower by about 20% for the series with 15% chalcedonite addition in relation to the series of concretes without the addition. The use of chalcedonite powder in concrete mixes seals the cement matrix.

It is recommended to extend the curing time of concrete because time has a positive effect on the parameters of compressive strength, capillary rise, and water absorption.

The aim of the laboratory research was to determine the influence of chalcedonite dust on the properties of concrete mixtures and the parameters of hardened concrete [31,32,33,34].

Currently, legal regulations and instructions do not provide guidelines on the amount of this additive that can be used. There is also no answer to the question of which rheological characteristics the use of chalcedonite dust in concrete mixtures improves or worsens. The article particularly focused on estimating the compressive strength of concretes with the addition of chalcedonite dust in three classes C30/37, C35/45, and C40/50, compared to concretes without this additive [35,36,37,38]. In addition, the concrete class was determined according to PN-EN 206-1:2016 for the series with the additive. The absorbency and capillary rise [12] were also examined after 28 days of maturation for each series with the addition of chalcedonite dust. The obtained research results will provide an answer to the possible use of waste chalcedonite dust in the production of concrete with specific parameters, which will enable the management of waste generated during the production of chalcedonite crushed aggregates. In the future, research and obtained results may be the basis for the development of instructions for the use of chalcedonite dust as an additive in concrete.

## 2. Materials and Methods

The aim of the laboratory research was to determine the parameters of three concrete mixes of class C30/37 (series 1), C35/45 (series 2), C40/50 (series 3) with the addition of chalcedonite dust, as well as three series without the addition of chalcedonite dust, C30/37 (series 1SW), C35/45 (series 2SW), C40/50 (series 3SW). Compressive strength, water absorption, and capillary suction were tested [32]. Mix designs were developed with a constant w/c ratio of 0.37 [33]. The following assumptions were made: exposure class-XF4, consistency of the mixture according to PN-EN 12350-8:2012-S3, [33]. Three series of laboratory tests were carried out on 21 cubic samples with a side length of 15cm. The test plan included testing for compressive strength after 2, 7, 14, 28, 56, and 90 days, water absorption, and capillary suction after 28 days of maturity, [34]. The concrete mixes were made from CEM I 42.5R cement. In the laboratory research, chemical admixtures were added to the mixture components in an amount of 0.2 ± 0.7% by weight of cement.

**Figure 1:**
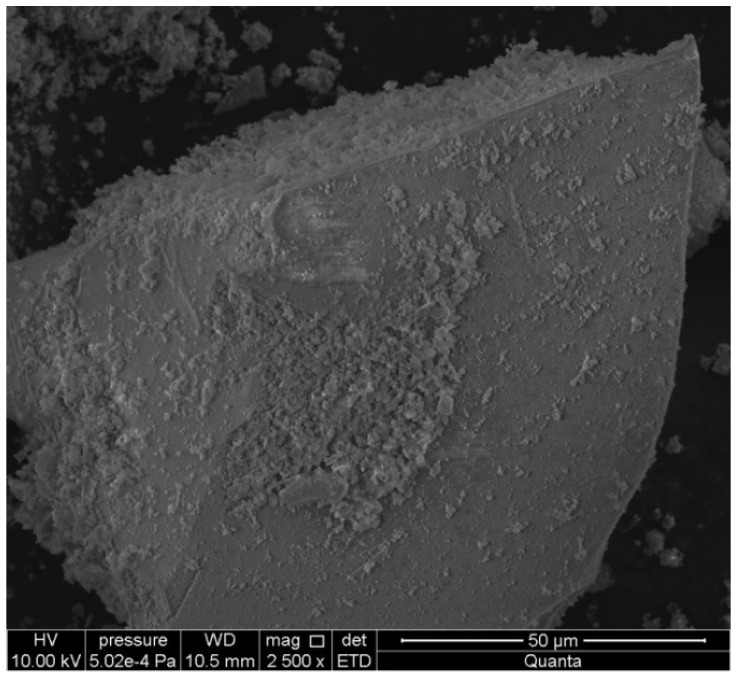
Microstructure of chalcedonite dust.

The chalcedonite dust used in laboratory tests is a waste material generated during the crushing of chalcedonite aggregate in the mine. It has the same mineral (chemical) composition as the aggregate it comes from. It has a negative impact on human health and can cause respiratory diseases (Figure 1). The dust consists mainly of quartz, accounting for 91.8% (Table 1). Analysis of the test sample also revealed the presence of moganite, a form of silica thatis difficult to detect but commonly occurs with chalcedony.

The particle size of chalcedonite meal was measured by laser diffractometer.

x_10_ = 0.28 µm x_50_ = 3.87 µm x_90_ = 25.53 µm SMD = 0.90 µm VMD = 9.50 µm

x_16_ = 0.44 µm x_84_ = 22.38 µm x_99_ = 34.99 µm S_V_ = 6.64 m^2^/cm^3^ S_m_ = 66,392.20 cm^2^/g

**Figure 2:**
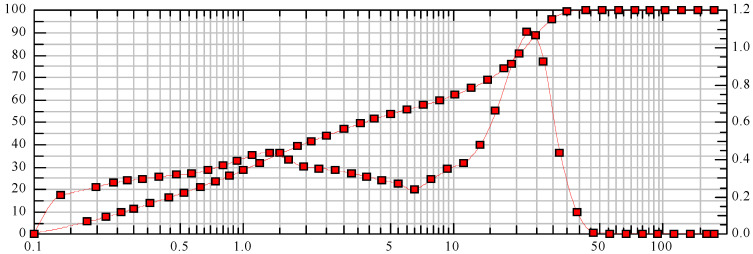
Particle size of chalcedonite powder.

The particle size distribution of the dust was established based on laboratory tests. The share of particles with a size up to 2 µm is 17.5%. In the range of 2 to 10 µm, it constitutes 22.5%. The largest share belongs to particles with a size of 10 to 40 µm, which is 42.5%. Moreover, 99% of the particles do not exceed a size of 72 µm (Figure 2).

Laboratory tests were performed in accordance with the composition in Table 2.

Laboratory tests were conducted using Portland cement CEM I 42.5R (Table 3) and a constant amount of chalcedonite dust, 15% of the cement mass. Sand with a fraction of 0/2 mm was used, which is a natural, fine-grained, river-origin aggregate with a stable grain composition and a high quartz content (>92%). The sand contains 55–80% of the fraction below 0.5 mm, which classifies it as one of the finest aggregates according to the PN-EN 12,620 standard.

Dolomite aggregate with fractions of 2/8 and 8/16 was used in the tests. It is a broken aggregate, and its main components are calcium and magnesium carbonate. The aggregate was used in accordance with PN-EN 12,620 “Aggregates for concrete” standard [21].

Tap water was used to prepare the planned concrete mixes in accordance with PN-EN 1008:2004 standard [11].

ISOLA BV plasticizing admixture was used, which is suitable for the production of monolithic, water-resistant, hydrotechnical concrete, as well as concrete exposed to chemical aggression. The action of this admixture improves the workability of the concrete mix at the same w/c ratio, does not affect the cement setting time, reduces water demand, and also increases the early strength of the concrete. The admixture should be used in an amount of 0.2 to 1.4% by weight of cement, depending on the expected plasticizing effect. The admixture was dosed directly into the mixer before adding the mixing water.

SikaControl^®^-4 WT (Sika, Warsaw, Poland) Concentrate admixture was used as a sealing admixture. It is an admixture with sealing and hydrophobic properties designed for ordinary and special concrete. It improves workability during compaction, water resistance, absorption, and capillary water absorption. The dosing rate is 0.1%–0.5% by cement mass, depending on the desired effect. In the tests, the admixture was added to the mixing water.

ISOLA LP A.E.A. 2.5 air-entraining admixture was used. It is used for the production of concrete exposed to changing atmospheric conditions and also increases the resistance to frost and de-icing agents. It was dosed into the wet components of the concrete mix [28,29].

The components of the concrete mix were mixed in the following order: coarse and fine aggregate with sand, then cement with the addition of chalcedonite dust, and finally, water was added. The plasticizing admixture was added to the dry ingredients before adding the mixing water, while the sealing admixture was added to the moist ingredients halfway through the addition of tap water.

The compressive strength was tested according to the standard PN-EN 206-1:2014 [11]. Cubic samples with a side length of 15cm were matured in water at a temperature of +18 ± 2 °C. The compressive strength was determined after 2, 7, 14, 28, 56, and 90 days of maturation. The strength test was carried out simultaneously on three samples [38,39,40,41].

Samples for testing water absorption were stored for 7 days in water at a temperature of +18 °C and then matured in air at a temperature of +18 °C. The samples were dried to a constant weight for 72 h in a climatic chamber. Then, the samples were placed in water up to half their height for one day. After this time, the water was filled up to +1cm above the sample height. The mass gain of the samples was measured every 24 h until two identical measurements were obtained [12].

The capillary rise was performed on samples with dimensions of 10 × 10 × 10 cm in accordance with standard PN-88/B-06250 [12]. The samples were matured in water at a temperature of +18 °C for 7 days after being formed. The samples were then removed and stored in a dry environment at a temperature of +18 °C. The mass gain was measured after 28 days of maturation. Before testing, the samples were dried in a climatic chamber at a temperature of +105 °C for 72 h. After this time, the samples were placed in water, and the mass gain was measured after 15 min, 30 min, 1 h, and 4 h from the time the samples came into contact with water. Subsequent measurements were taken every 24 h. During the testing, the samples were submerged in water to a depth of approximately 3 mm. The sides of the samples were isolated to prevent uncontrolled moisture uptake from the environment.

## 3. Results

Rheological tests of the fresh concrete mix were performed. The volumetric density, consistency of the concrete mix using the cone drop method, and the air content in the concrete mixes were examined (Table 4 and Table 5). The consistency class of all tested mixes was S3.

**Figure 3:**
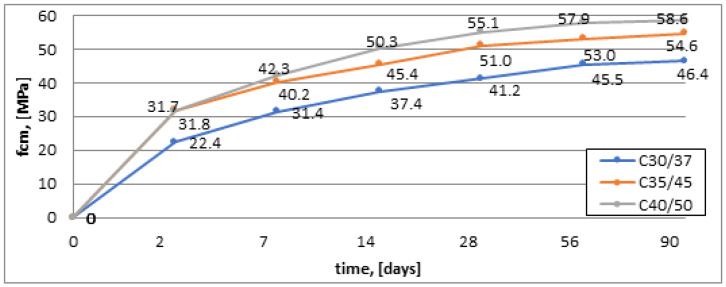
Increase in compressive strength for concretes with the addition of dust (MPa).

**Figure 4:**
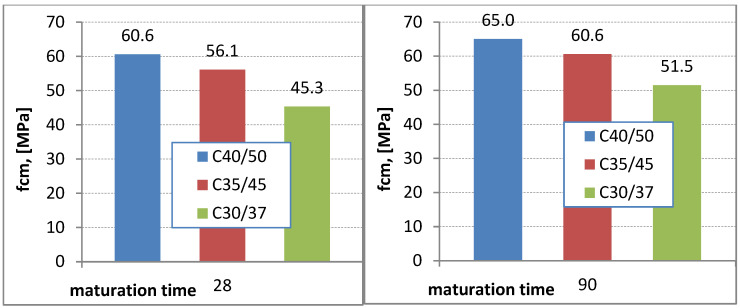
Increase in compressive strength for concretes without additive (MPa).

The compressive strength gain chart shows that series 3 (C40/50) exhibits the highest compressive strength. Compared to series 1, the strength gain is 33.3% higher after 28 days of maturation and 28.6% higher after 90 days of maturation. From the analysis of Figure 3 and Figure 4, it can be inferred that the highest compressive strength gain was observed for concrete samples without the addition of chalcedonite dust. Comparing the series with and without dust addition, for example, (series 1 and series 1SW), it can be noticed that concretes containing dust in their composition exhibit an average decrease in compressive strength of 10% after 28 and 90 days of maturation (Figure 3 and Figure 4).

All obtained compressive strength results (for the series with the addition of dust, Figure 3) were checked against the concrete strength class. Method A was used. The obtained compressive strength results were classified into designed concrete classes (Table 6). The conditions were met despite a decrease in compressive strength by an average of about 10% compared to the series without this addition.

**Figure 5:**
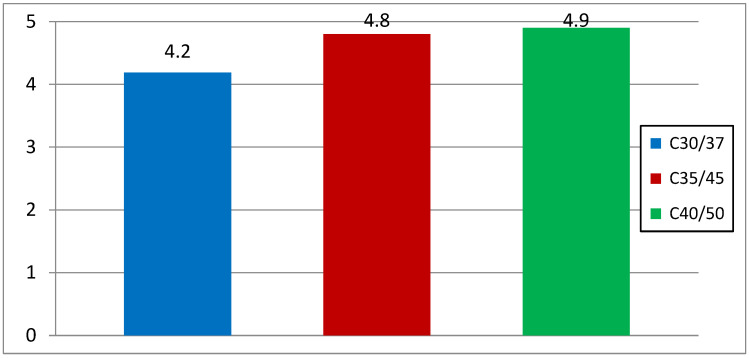
Weight gain of concrete with dust in the saturation test after 28 days of maturation, [%].

Comparing the results of water absorption by concrete specimens (Figure 5) during the water permeability test, comparable results were obtained below 5%. The PN-88/B-06250 standard [12] states that the water absorption of concrete should not exceed 5% for concrete exposed directly to atmospheric factors. Therefore, these concrete classes can be used in an environment exposed to water.

**Figure 6:**
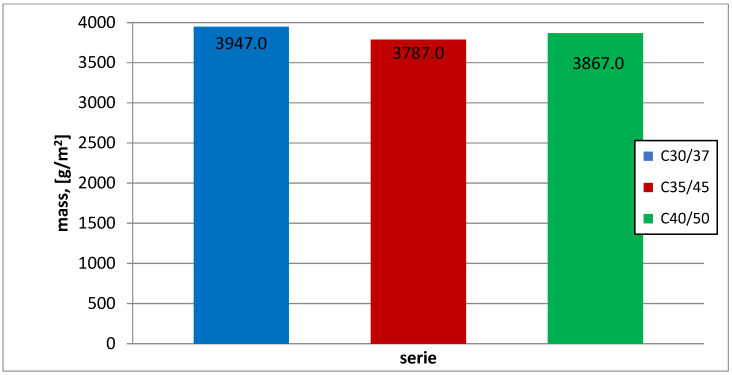
Weight gain of concrete specimens with dust in capillary rise test after 28 days of maturation (g/m^2^).

In the study of capillary absorption of concrete samples (Figure 6) of the three classes under consideration, we also obtained comparable results of mass gain after 28 days of maturation. Comparable results of laboratory tests were obtained by introducing dust into the concrete mixture in a comparable amount to the cement. In this case, the dust sealed the structure of the concrete.

After obtaining the results of laboratory tests, such as compressive strength, water absorption, and capillary absorption, relationships between these three parameters after 28 days of maturation were established.

**Figure 7:**
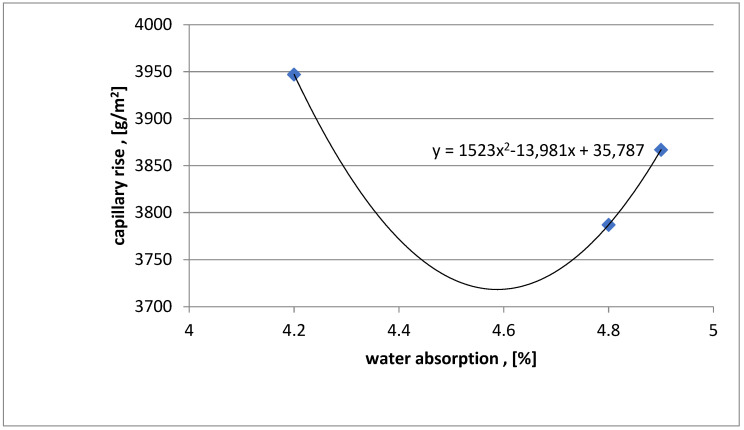
Polynomial relationship between water absorption and capillary rise after 28 days of maturation for concretes with dust.

In Figure 7, the water absorption results for the three concrete series with dust were introduced on the *x*-axis, and the capillary absorption results were introduced on the *y*-axis for the three series with added chalcedonite dust, which wasexamined after 28 days of maturation. Thanks to these results, a polynomial relationship between water absorption and capillary absorption for the three series of tested concrete with 15% chalcedonite dust was estimated.

**Figure 8:**
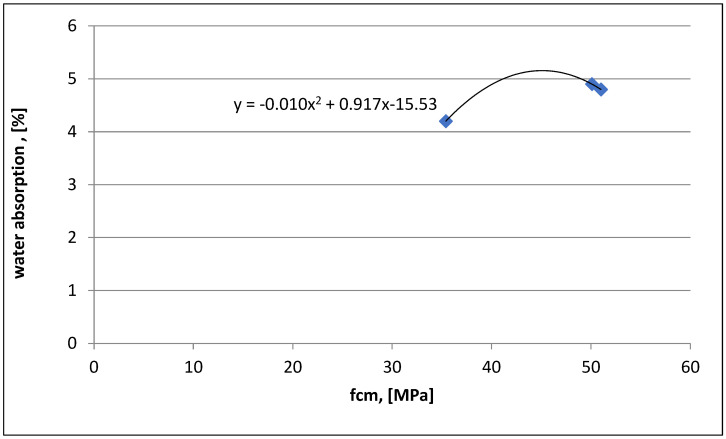
Polynomial relationship between water absorption and compressive strength after 28 days of maturation for concretes with dust.

In Figure 8, a polynomial relationship between the water absorption and compressive strength was estimated for three tested series of concrete with the addition of dust. The compressive strength results after 28 days of maturing were marked on the *x*-axis, and the water absorption results for concrete classes C30/37, C35/45, and C40/50 were marked on the *y*-axis. The water absorption and compressive strength tests were performed on samples after 28 days of maturing. The results were analyzed using Excel software, (Microsoft, Poland).

**Figure 9:**
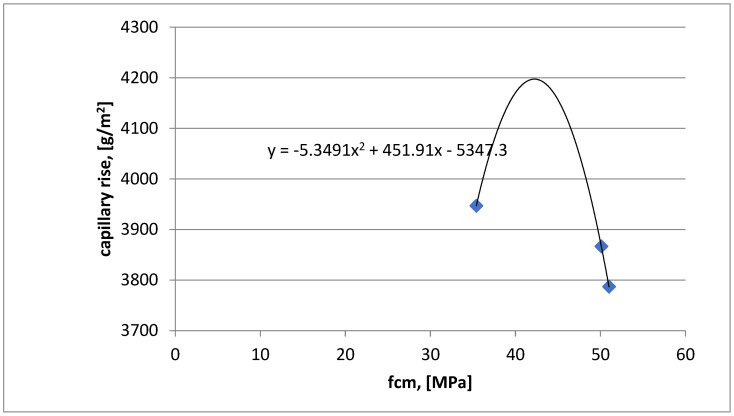
Polynomial relationship between capillary rise and compressive strength after 28 days of maturation for concretes with dust.

Figure 9 shows a polynomial relationship between water absorption and capillary rise. The relationship was estimated for the results of three series with the addition of dust. The samples were tested after 28 days of curing. The relationship was obtained from calculations in the Excel program.

After determining the mutual dependencies from any class of concrete C30/35, C35/37, and C40/50, instead of specifying individual parameters, one can determine one characteristic of hardened concrete and estimate the remaining ones based on the equation. This is a faster and less laborious process than conducting individual laboratory tests.

**Figure 10:**
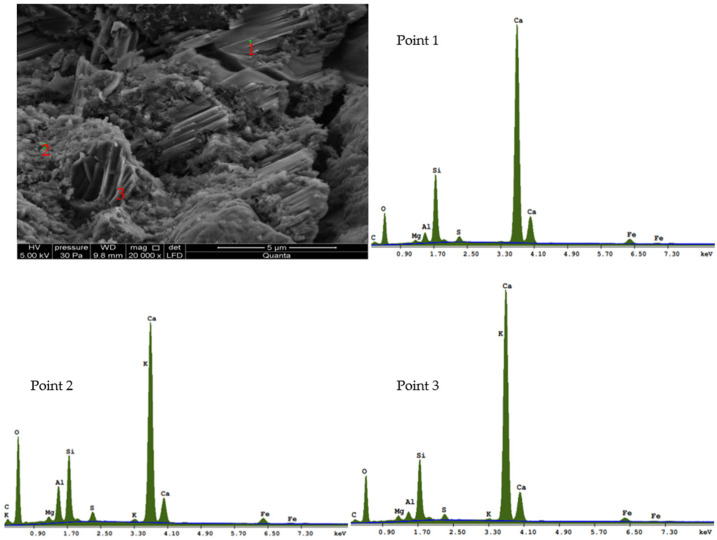
X-ray examination with chalcedonite dust at 1, 2, and 3 points for C35/45 series.

The examination of the concrete microstructure for the C35/45 series with chalcedony dust, conducted with a scanning electron microscope, showed that the additive was active in hydration. Confirmation should be sought in the lack of clear boundaries between the dust and the C-S-H fraction (Figure 10). The addition of 15% of dust indicates beneficial, although weak pozzolanic properties, as confirmed by laboratory test results for compressive strength after 28 and 90 days of curing.

## 4. Discussion

After laboratory tests of concretes of class C30/37 (series 1), C35/45 (series 2), C40/50 (series 3) with the addition of chalcedonite dust, and three series without the addition of chalcedonite dust C30/37 (series 1SW), C35/45 (2SW series), C40/50 (3SW series) it should be stated that the problem raised in the article—will the compressive strength after 28 days be high enough to qualify the given concretes to the designed classes? The article checks the class of concrete according to PN-EN 206+A1: 2016 for the series with the addition of dust. The conditions were met, despite obtaining a decrease in compressive strength of about 10% on average compared to the series without this additive. In the literature, the researchers published the results of compressive strength after 28 days of maturation, where a decrease in compressive strength was noted, depending on the amount of chalcedonite dust added. For 5% of the added additive, the strength is maintained. The addition of 20% of dust in relation to the mass of cement causes a decrease in compressive strength by 15%, and 15% of dust is a decrease of 10%. When designing the research for this article, we assumed that if we wanted to maintain the assumed classes of concrete, we could not add more than 15% of the dust in relation to cement. After analyzing the literature and carrying out the previous tests, it can be concluded that chalcedonite dust does not have full pozzolanic properties. The additive, which is distinguished by pozzolanic properties, is added in the amount of 20% of the cement mass, and the concrete does not experience a decrease in strength. The second aspect taken into account in the article was the problem of managing heaps of tailings in the mine. The possibility of its use in concrete mixes was checked. The answer is yes, only under the condition of replacing cement up to a maximum amount of 15% of its weight. After exceeding this amount, there will be a decrease in compressive strength even after a long curing time of 56 or 90 days. You can expect a lower strength class and, thus, different use of the designed concrete.

The addition of chalcedonite dust reduces the weight gain of samples in contact with water in the water absorption test. A significant decrease in mass gain can be observed in the case of concrete with the addition of 15% chalcedonite dust;this decrease is 21% compared to the reference concrete. Concrete with 10% of chalcedonite dust reduces the weight gain of concrete by about 11%. The use of chalcedonite dust in concrete mixes seals the cement matrix.

In the capillary rise test, it can be seen that the weight gain is comparable tothe tested series, in which chalcedonite dust was used in the amount of 15%. From the literature, we learn that by adding dust in the amount of 5%, 10%, and 15%, the weight gain is about 35–40 kg/m^2^ after 28 days of maturation. The differences are not too big;it is due to the fact that dust has a positive effect on concrete, making it more airtight.

Considering economic and environmental factors, it is advisable to use an additive to concrete in the form of chalcedonite dust in the amount of up to 15%. The use of chalcedonite dust as an additive to concrete could help solve the problem of storage and management of waste from the production of chalcedonite aggregates. This value of 15% is the maximum value that can be added to concrete without impairing its parameters to such an extent that the concrete will then be classified as a class lower than the designed one.

## 5. Conclusions

The results of the study provide the basis for the following conclusions:

1. Compressive strength: after 28 days of maturation, all three tested series of C30/37 (labeled as 1SW), C35/45 (labeled as 2SW), and C40/50 (labeled as 3SW) with the addition of chalcedonite dust achieved the assumed strength classes. The components of concrete mixtures were correctly designed. The addition of chalcedonite dust to the concrete mixture did not result in a decrease in compressive strength to such an extent that the analyzed series did not meet the normative requirements regarding concrete classes according to PN-EN 206+A1:2014, [11]. Comparing the compressive strength of the series with the addition of chalcedonite dust to those without it resulted in an average strength reduction of 10%.
2. Water absorption: the obtained results indicate water absorption below 5%. This means that the concretes made in laboratory conditions (precise dosing of components, known quality and strength parameters) are characterized by water absorption not exceeding 5% for series C30/37 (1SW), C35/45 (2SW), C40/50 (3SW) with the addition of chalcedonite dust.
3. Capillary absorption: the C30/35 (1SW), C35/37 (2SW), and C40/50 (3SW) concrete classes, with the addition of chalcedonite dust, achieved similar values.
4. Dependencies of compressive strength on water absorption or capillary absorption, as well as water absorption on capillary absorption, were developed for the three tested concrete classes C30/37 (1SW), C35/45 (2SW), C40/50 (3SW) with the addition of chalcedonite dust after 28 days of maturation.
5. Based on the obtained laboratory test results, chalcedonite dust can be used as an additive to concrete in an amount of 15% of the cement mass. The use of chalcedonite dust as an additive to concrete could help solve the problem of storing and managing waste from chalcedonite aggregate production in Poland.
6. Consideration should be given to attempting to develop general guidelines for the use of this additive in concrete mixtures in order to manage billions of tons of waste from broken chalcedonite aggregate mines. Disposing of waste in this way could help people protect their health from the adverse effects of inhaling airborne dust.

## Figures and Tables

**Table 1:** Chemical composition of dust chalcedonite [27].

Chemical Compound	Elemental Content, [%]
F	0.06
NA_2_O	0.09
MgO	0.15
Al_2_O_3_	3.04
SiO_2_	91.8
P_2_O_5_	0.04
SO_3_	0.05
K_2_O	0.42
CaO	0.45
TiO_2_	0.11
Fe_2_O_3_	1.28
ZrO_2_	0.01
BaO	0.04

**Table 2:** Constituents of concrete mixtures.

Component, (kg/m^3^)	Serie 1	Serie 2	Serie 3	Serie 1SW	Serie 2SW	Serie 3SW
Cement CEM I 42.5R	420	440	460	483	506	529
Chalcedonite dust	63	66	69	---	---	---
Water	156	164	173	156	164	173
Chalcedonite sand 0/2 mm	680	600	600	680	600	600
Dolomit 2/8 mm	600	500	530	600	500	530
Dolomit 8/16 mm	650	700	680	650	700	680
Plasticizing admixture	1.82	1.82	1.82	1.79	1.79	1.79
Sealing admixture	4.1	4.1	4.1	4.0	4.0	4.0
Aerating admixture	2.72	2.72	2.72	2.7	2.7	2.7

**Table 3:** Information on basic properties of the cement [9].

No.	Cement Type	Chemical Composition, %					Blaine	Resistance, MPa	
		SiO_2_	CaO	MgO	Fe_2_O_3_	Al_2_O_3_	cm^2^/g	R_2_	R_28_
1	CEM I 42.5R	19.49	62.3	2.08	3.25	4.75	4053	32.0	53.0

**Table 4:** Volumetric density of the tested concrete mixtures (own elaboration).

Series Name	Volumetric Density, (kg/m^3^)
series 1	2374
series 2	2392
series 3	2408
series 1SW	2414
series 2SW	2437
series 3SW	2493

**Table 5:** Air content of the tested series of concrete mixtures (own elaboration).

Series Name	Air Content, (%)
series 1	7.0
series 2	7.2
series 3	7.3
series 1SW	6.9
series 2SW	7.0
series 3SW	7.4

**Table 6:** Verification of the class of concrete according to EN 206-1:2016 for the series with dust addition.

Series	Conditions	Value	Criterion fulfilled
Criterion 1	fcm ≥ fck + 4	41.2	YES
Criterion 2	fci ≥ fck−4	41.1	YES
Conclusions: The tested concrete meets the compressive strength requirements for class C30/37 according to PN-EN 206-A1:2016			
		Value	Criterion fulfilled
Criterion 1	fcm ≥ fck + 4	51.0	YES
Criterion 2	fci ≥ fck − 4	50.2	YES
Conclusions: The tested concrete meets the compressive strength requirements for class C35/45 according to PN-EN 206-A1:2016			
		Value	Criterion fulfilled
Criterion 1	fcm ≥ fck + 4	55.1	YES
Criterion 2	fci ≥ fck − 4	54.7	YES
Conclusions: The tested concrete meets the compressive strength requirements for class C40/50 according to PN-EN 206-A1:2016			

## Data Availability

Not applicable.
